# The Impact of Alternative Protein Sources on Gut Microbiota: Limitations of the Current Understanding

**DOI:** 10.3390/foods15071145

**Published:** 2026-03-26

**Authors:** Lara Costantini, Nicolò Merendino

**Affiliations:** Department of Ecological and Biological Sciences, Tuscia University, 01100 Viterbo, Italy

**Keywords:** microbiota, alternative proteins, insects, algae, fungi

## Abstract

While the effects of carbohydrates and fibers on the gut microbiota are well established, the influence of proteins has only recently been explored. In addition, whereas the impacts of conventional protein sources remain partially defined, evidence is even more limited for alternative sources, such as insects, algae, and fungi. This commentary highlights the critical knowledge gaps in the context of the impact of alternative protein sources on gut microbiota, which need future investigation. This understanding becomes even more critical when considering that many of these sources are regarded as novel foods by large segments of the global population. Emerging findings suggest that differences in protein structure and digestibility may increase the protein content in the lumen, which in turn could enhance microbial protein fermentation and the production of poorly characterized metabolites. Moreover, the gut microbiota may be less adapted to metabolizing and managing such novel substrates if they were not previously part of the habitual diet. Therefore, well-designed clinical trials are urgently needed to understand the real potential effects of the alternative proteins on gut microbiota.

## 1. Introduction

A strong body of scientific evidence has linked the Mediterranean diet to increased survival and a reduced risk of non-communicable diseases in several population cohorts worldwide [1]. The protective effect related to the Mediterranean diet is attributed to the daily intake of plant-based foods, together with a balanced and moderate inclusion of animal-based foods (i.e., fresh meat and fish), while the consumption of processed meats should be limited [2]. The WHO/FAO/UNU Dietary Guidelines recommend a protein intake of 0.8 g/kg/day for healthy adults to meet their nutritional requirements (48–56 g/day considering an average weight of 60–70 kg, respectively) [3]. However, in recent years, a decrease in adherence to the Mediterranean diet has been documented [4] in association with higher protein consumption, at 76.6 g/day per capita, which is expected to increase to 79.8 g/day by 2033 [5]. At the same time, it is predicted that by 2050, the world population will reach nearly 9.7 billion people [6], requiring almost 119% increase in the production of edible crops [7]. By 2050, it is also worth noting that the increase in atmospheric CO_2_ is projected to lead to a decline in the protein content of staple crops such as rice, potatoes, wheat, and barley by 6%, potentially placing approximately 150 million individuals at risk of protein deficiency [8]. In this context, new protein sources with greater sustainability have been analyzed in the literature with the aim of increasing the protein content of diets across the global population. Although the replacement with plant-based proteins is the most conventional approach and aligns with the Mediterranean diet, they often show limited consumer acceptance, particularly in the case of legumes [9]. As a result, plant-based analogs of meat, fish, eggs, and dairy products have been developed to mimic the sensory characteristics of these foods. However, such products typically contain long lists of ingredients and food additives aimed at improving their sensory and technological properties, which may ultimately compromise their overall nutritional profile [10]. Therefore, other unconventional sources, such as insects, algae, and fungi, have also been explored, even in geographic regions with no prior history of their consumption. Indeed, these alternative sources have been promoted as the most promising options for ensuring nutritional security while minimizing the adverse impacts associated with animal protein systems [11].

In recent years, the study of human microbiota has become increasingly important in understanding many physio-pathological conditions and in relating them to various external environmental factors, even if the causal relationship between gut microbiota and the development of pathological conditions remains a matter of ongoing debate. It is still unclear whether a disease-associated microbial configuration, commonly referred to as dysbiosis, represents a causal factor in disease onset or rather a consequence of the pathological state [12]. Nevertheless, diet is widely acknowledged as one of the main drivers shaping gut microbiota composition, as dietary components provide substrates that can be metabolized by both the host and intestinal microorganisms. While numerous studies and evidence are present in relation to the fermentation of carbohydrate matrices by the intestinal microbiota, much less is known about other nutritional components, like proteins [13]. In the past, the concept that our symbiotic microorganisms not only utilize dietary fiber but also require and metabolize a wide range of other nutritional molecules was largely overlooked. Like the human host, these microorganisms require a balanced supply of such nutritional compounds, which could be described as a “correct prebiotic diet” [14]. Therefore, a deficiency in the necessary nutritional components leads to a decline in microbial diversity and abundance, while their excess results in the accumulation of fermentation-derived metabolites within the host, thereby triggering biochemical and metabolic perturbations in both the host and the commensals. This commentary aims to provide an integrated overview of current insights into how different protein sources, both conventional and alternative, influence the gut microbiota, and to identify the critical knowledge gaps that should be addressed through future research. This understanding is especially crucial in the context of alternative protein sources, many of which are regarded as novel foods by large segments of the global population.

## 2. Conventional Protein Sources’ Impact on Gut Microbiota

Approximately 12–18 g of undigested dietary and endogenous proteins reaches the intestinal lumen daily, where they are metabolized by the gut microbiota [15]. As the first step of microbial metabolism, undigested proteins are hydrolyzed by extracellular proteases and peptidases. The resulting amino acids and peptide residues are then transported into bacterial cells and directed toward anabolic pathways as building blocks or enter catabolic routes for further degradation [16]. Among the earliest metabolic steps are transamination and deamination reactions, which may take place through oxidative, reductive, or coupled mechanisms (e.g., Stickland reaction) [13]. These pathways give rise to a complex array of metabolic end products, including short-chain fatty acids (SCFAs), branched-chain fatty acids (BCFAs), hydrogen sulfide (H_2_S), polyamines, phenols, indoles, ammonium (NH_4_^+^), and numerous other metabolites that remain to be identified [15]. Evidence in the literature showed that diets enriched in amino acids and related nitrogenous substrates (e.g., choline, betaine, l-carnitine, tyrosine, phenylalanine, and tryptophan) have been associated with increased microbial production of uremic toxins, such as phenylacetic acid, p-Cresyl sulfate, trimethylamine-N-oxide, asymmetric dimethylarginine, indoxyl sulfate, and indole acetic acid [17]. The pathogenicity of uremic toxins lies in the concept of the “multidirectional network” that exists between the microbiota and the host. Indeed, these compounds can be metabolized into different, and sometimes more harmful, molecules either by other microbial members within the same community, through cross-feeding interactions, or by migrating to other host organs where they undergo further chemical modifications [18]. A well-established example in the literature is trimethylamine, generated by microbial metabolism of choline and subsequently oxidized to trimethylamine N-oxide (TMAO) by hepatic flavin-containing monooxygenases. Circulating TMAO concentrations have been consistently linked to an increased risk of cardiovascular events [19]. Although the underlying mechanisms remain unclear, emerging evidence indicates that uremic toxins exacerbate oxidative stress by increasing reactive oxygen species (ROS) production and impairing antioxidant defenses [20]. The production of uremic toxins is tightly influenced by multiple factors, including the digestibility of dietary proteins. Indeed, digestibility depends on the structural matrix of the food and its accessibility to proteases, the presence of non-protein and anti-nutritional factors that may slow protein digestion, and, last but not least, the amount and type of protein consumed [16]. However, the relative contribution of these factors remains debated. A comprehensive review by Ma et al. [21] outlines how animal- and plant-derived proteins differentially influence the gut microbiome and host health, considering their nutritional quality and digestibility. Despite lower digestibility and a more limited amino acid profile, plant proteins contain bioactive components that favor the enrichment of beneficial taxa, such as *Bifidobacterium*, *Lactobacillus*, and *Akkermansia*, and are generally associated with improved metabolic outcomes. By contrast, animal-derived proteins, while providing a more complete amino acid profile, exert effects that are highly dependent on their dietary source, with notable differences observed among red meat, dairy, and fish. Their potential benefits are also strongly modulated by intake levels and processing methods [21]. In parallel, the study by Awan et al. [22] in mice found that brown rice proteins exhibited poor digestibility, whereas soy and pea proteins were efficiently processed by the host. At the same time, animal proteins, which are generally considered highly digestible, such as those from egg white, were detected in higher amounts within the intestinal lumen compared with soy and pea proteins. Collectively, the authors conclude that the metabolic fate of dietary proteins within the gut microbiota is more shaped by individual protein sources and molecular composition than by the simple animal-plant dichotomy [22]. In support of these findings, Blakeley-Ruiz et al. [23] demonstrated that different dietary protein sources can reshape the metabolic functions of the gut microbiota by altering the composition of bacterial populations. Among all protein sources examined in their study (i.e., soy, casein, rice, yeast, pea, and egg white), egg white protein promoted the enrichment of bacterial taxa possessing amino acid deaminating enzymes and ureases, which are associated with degradation of the intestinal mucus barrier. Notably, this effect occurred independently of the amount of protein administered [23]. At the same time, a recent meta-analysis of 15 randomized controlled trials examined the effects of different protein sources while also accounting for dietary fiber intake. The analysis revealed that, overall, neither total protein intake nor protein source substantially altered gut microbiota composition, although higher protein consumption appeared to modulate proteolytic metabolic functions within the microbiota. However, fiber intake emerged as a key determinant of the overall impact, with high-protein/low-fiber diets leading to significantly higher circulating TMAO levels compared with high-protein/high-fiber regimens [24].

Taken together, these lines of evidence highlight the need for further investigation into conventional protein sources through large-scale, long-term prospective population studies. Most current data are derived from animal or *in vitro* experiments, in which it remains challenging to accurately replicate the complex dietary environments, physiological contexts, and long-term effects observed in humans. Moreover, the protein sources used in studies are often purified or isolated preparations that fail to capture the compositional and structural complexity of whole food matrices, thereby leaving the impact of conventional protein sources on human health an unresolved issue.

## 3. Alternative Protein Sources and Microbiota: What We Know and What We Should Know

We are living in a historical period in which, while low-income countries are shifting their diets from plant-based to animal protein sources, high-income countries are witnessing the introduction of novel alternative protein sources into the food system to replace animal proteins and meet the rapidly increasing global protein demand projected for 2050 [25]. Indeed, a variety of novel alternative protein sources have been developed with the aim of replacing animal-derived proteins, which are considered both healthier and more sustainable. These include insects, algae, and fungi. However, Western high-income populations have little or no historical precedent for the consumption of such alternative protein sources. Therefore, their effects on the Western populations remain largely unknown, especially considering that proteins from such sources often exhibit unique structural properties [26]. For example, although insect proteins exhibit considerable interspecies variability due to their diverse origins, muscle and hemolymph proteins are predominant across most insects. Algae harbor specialized functional proteins, known as phycobiliproteins, that are essential for their photosynthetic activity and survival. Fungal proteins commonly assemble into elongated filamentous networks that need to preserve cellular architecture. Moreover, both fungi and insects possess lectins, although these proteins exhibit markedly distinct structural features across taxa [26]. This is particularly relevant considering that these sources are often rich in protein, in some cases providing substantially higher protein contents than conventional sources. Indeed, for example, among algae, *Spirulina* sp. and *Chlorella vulgaris* have a protein content up to 70% and 62%, respectively [26,27], similar values or slightly higher than 70% were found for the insects *Acheta domesticus* (house cricket), *Tenebrio molitor* (yellow mealworm), and *Locusta migratoria* (migratory locust) [28], and among microorganisms, Quorn, from *Fusarium venenatum,* showed protein values between 45% as dry weight [29].

It should also be noted that, as discussed above, the higher protein content of these alternative foods may increase the absolute amount of protein reaching the intestinal lumen. Moreover, a proportionally greater accumulation of alternative protein within the intestinal lumen may occur with increased levels of poor digestibility. For example, cricket and mealworm showed lower *in vitro* protein digestibility (63.6% and 69.5%, respectively) than milk, soy, and wheat (86.1–90.8%, 85.1%, and 82.3%, respectively) [30]. Indeed, insects, algae, and microbial proteins also contain higher levels of anti-nutritional factors than conventional animal proteins. These structural and biochemical barriers limit enzymatic proteolysis and absorption [31]. Therefore, given the potentially high amounts that could reach the intestinal lumen, it would be crucial to elucidate their impact on the gut microbiota, even if to date, our understanding remains extremely limited. Indeed, although there are some preclinical studies, only one clinical trial has examined gut microbiota composition and metabolism following insect consumption in populations where insects are considered novel foods. In the pilot cross-over study by Stull et al. [32], consumption of 25 g/day of *Gryllodes sigillatus* for 14 days resulted in a 5.7-fold increase in *Bifidobacterium animalis*, accompanied by a concomitant 3- to 4-fold decrease in *Limosilactobacillus reuteri*. Overall, a significant reduction in excreted acetate and propionate was observed. The authors proposed that these findings might reflect the inability of the study population, unaccustomed to insect consumption, to effectively metabolize novel fiber sources such as chitin [32]. Indeed, although chitin has traditionally been regarded as an indigestible, insoluble dietary fiber, accumulating evidence indicates that certain human cells express chitinases and chitinase-like proteins [33] and that chitinase activity can be detected in the gastric environment [34]. These findings suggest a previously unrecognized capacity for endogenous chitin degradation in humans and highlight the need for future studies to determine whether individuals who habitually consume insects might be more enzymatically equipped to digest insect-derived chitin. However, this pilot study did not account for the potential impact of the higher protein content provided by the insect-based meal (i.e., more than twice that of the control meal) on human gut microbiota composition, nor did it assess whether the observed differences could be attributed to the increased ammonia load or other protein-related metabolites. In parallel, preclinical *in vitro* and animal studies, despite conducted in different species, administering different types of insects, and diverse intervention and analytical approaches, consistently indicate that proteolytic fermentation by gut bacteria promotes increased production of BCFAs and ammonia [35].

Similarly, only limited information is available for microalgae, confined to preclinical studies, while specific clinical trials investigating the impact of these alternative protein sources on the gut microbiota are scarce. In preclinical studies conducted in rats and mice, *S. platensis* supplementation consistently increased commensal microbiota populations and SCFAs production, leading to an overall improvement in systemic inflammatory status [36]. Only a recent randomized, double-blind, placebo-controlled trial has assessed the impact of *S. platensis* on intestinal permeability and oxidative stress in patients with constipation-dominant irritable bowel syndrome, reporting significantly reduced levels of zonulin (i.e., a marker of increased permeability) and malondialdehyde (a marker of oxidative stress) [37]. However, clinical studies investigating the impact of *S. platensis* on the composition of the human gut microbiota and its associated protein metabolites are currently lacking. By contrast, a randomized, double-blind, placebo-controlled crossover trial in Japanese patients with constipation investigated the effects of *C. vulgaris* on the gut microbiota and metabolome [38]. The study did not reveal statistically significant differences in overall microbiome or metabolome profiles but identified increased levels of two specific metabolites (i.e., azelaic acid and dicarboxylic acids) which the authors attributed to the microbial metabolism of *C. vulgaris* fatty acids in the human gut [38]. However, it should be noted that the Japanese population examined in this study habitually consumes seaweeds, a dietary habit that may have “trained” the gut microbiota to utilize such alternative nutrient sources over time. Indeed, the gut microbiota of Japanese individuals is known to harbor specific carbohydrate-active enzymes (i.e., CAZymes), such as porphyranases and agarases, which enable the digestion of seaweed-derived polysaccharides, enzymatic activities that, for example, are absent in North American populations [39]. Studies have shown that these enzymatic capabilities were acquired by the Japanese gut microbiota through gene transfer from marine bacteria, likely originating from non-sterile foods such as sushi [39]. For this reason, the concept of intra-host evolution has gained increasing attention in recent years, referring to the observation that within-host strains undergo gene loss and acquisition, together with nucleotide variants, which can reach high frequencies within a matter of months [40].

Future challenges will therefore include elucidating the impact of microalgae on populations with distinct dietary habits, as well as investigating whether “adapted” populations have evolved specific mechanisms for detoxifying potentially harmful metabolites arising from protein digestion.

Few and conflicting findings have also been reported regarding the impact of mycoproteins (e.g., Quorn) on the human gut microbiota. Indeed, whereas the preclinical study by Cherta-Murillo et al. [41] failed to detect *in vitro* fermentation of mycoproteins by the human microbiota within 24 h, Colosimo et al. [42] reported increases in SCFAs and significant rises in *Bacteroides* after 72 h. These findings suggest that the human gut microbiota may require a longer adaptation period to ferment the fiber structures characteristic of mycoproteins (i.e., β-glucans + chitin). However, these preclinical studies did not examine the contribution of the protein fraction of mycoproteins to the generation of additional protein-derived microbial metabolites, such as uremic toxins. Instead, in the recent paper of James et al. [43], a preclinical *in vitro* study compared the effects of multiple dietary protein sources (i.e., whey, fish, milk, soya, egg, pea, and mycoprotein) on the human microbiota. Mycoprotein fermentation resulted in elevated levels of isobutyrate and valerate, but also of phenol, p-cresol, and ammonia, metabolites associated with impaired epithelial barrier function *in vitro* and promoted a more proteolytic bacterial community that included members of the *Bacteroides* and *Clostridium* genera [43]. However, clinical evidence remains limited. In the only available trial by Farsi et al. [44], substantial portions of red meat (i.e., 240 g/day) were replaced with equivalent portions of mycoprotein for two weeks following a cross-over protocol. Mycoprotein supplementation elicited significant increases in SCFAs, but these were mainly attributable to increases in fiber intake compared with the habitual diet (+16.99 g/day), as well as the observed reduction in BCFAs was driven by a parallel decrease in protein consumption in comparison to the volunteers’ habitual diet (–11.41 g/day). Moreover, after mycoprotein consumption, although a significant decrease in *p*-cresol sulphate and nitroso compounds was observed, the levels of two genotoxic metabolites, apocholic acid and 7-ketodeoxycholic acid, did not differ significantly from those measured during the red-meat phase. Thus, although the study reported certain benefits of mycoprotein intake, including a significant reduction in ammonia, further comparative trials involving healthier protein sources are required to clarify the magnitude and relevance of these effects [44].

**Figure 1:**
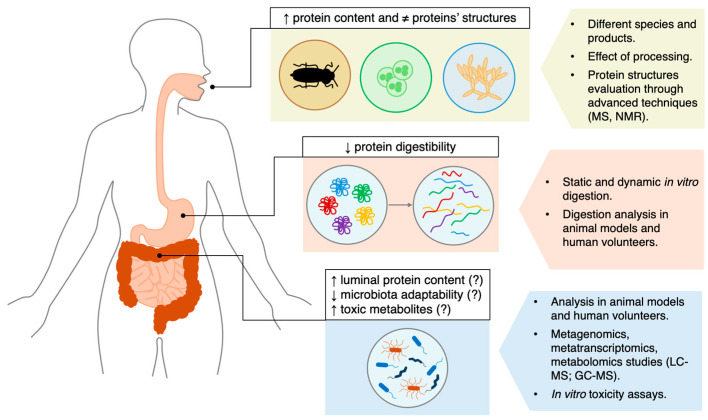
Limitations of current understanding on the impact of alternative protein sources on the gut microbiota. Insects, algae, and fungi contain higher percentages of protein and exhibit protein structures that differ markedly from those of conventional sources. Early evidence also indicates reduced digestibility, driven by the presence of anti-nutritional factors and by structural and biochemical barriers. Preliminary data—largely from *in vitro* studies—suggest that these features may increase the luminal protein load that comes into contact with the gut microbiota, which in turn may be less adapted to metabolizing such novel substrates if they were not previously part of the habitual diet. Moreover, the high protein content of these alternative sources could enhance microbial protein fermentation and the production of potentially harmful metabolites. The right panels outline priority directions for future research to overcome current limitations.

Previous studies have indicated that the microbiota of centenarians is better adapted to physiological changes, exhibiting the capacity to provide alternative metabolic solutions that confer long-term advantages. Such a resilient microbiota may therefore be better able to maintain eubiosis and support overall health [45]. So, considering the above, could the introduction of alternative protein sources pose challenges for adaptation and cause perturbations in the human microbiota, given that the dietary shift is occurring over a relatively short time span? Also considering the popularity of such diets and the high protein content of these alternative sources, might this lead to excessive protein intake and consequently increased proteolytic metabolism? It should also be considered that the individual health status and genetic background might differentially influence the microbiota response, resulting in distinct metabolic outcomes. Future research will need to address these potential issues, and further clinical studies will be essential to elucidate any unintended effects (Figure 1).

In conclusion, beyond the aspects discussed above, one of the major challenges in studying the impact of alternative proteins on the gut microbiota is the lack of human intervention trials and the limited evaluation of the production of potentially harmful secondary metabolites by the microbiota. In this regard, as a priority, a first study should consist of a crossover clinical trial in volunteers not routinely consuming such alternative sources, comparing protein isolates from alternative and conventional sources at equivalent doses, with assessment of fecal microbiota and metabolomic profiles. Subsequently, further studies should be focused on the characterization of fecal metabolites using advanced analytical techniques, such as mass spectrometry and nuclear magnetic resonance spectroscopy, as well as studies integrating metagenomics, metatranscriptomics, and metabolomic analyses could help to shed light on the magnitude of this impact (Figure 1). In addition, it should be emphasized that the preliminary analyses have so far involved only a limited number of species and types of alternative protein sources and did not take into account the potential effects of processing. Therefore, further analyses will also be required at this initial stage to determine whether the high protein content and the diversity of protein structures are maintained under different conditions. Subsequently, static and dynamic *in vitro* digestion analyses, as well as *in vivo* studies in animal models and human volunteers, may be useful to confirm whether reduced protein digestibility is also observed across other conditions (Figure 1).

## Data Availability

No new data were created or analyzed in this study.
